# Prenatal Diagnosis of Recurrent Fetal Intracranial Hemorrhage and a Maternally Inherited COL4A1 Variant of Uncertain Significance: A Case Report With Maternal Reverse Phenotyping

**DOI:** 10.1002/ccr3.73525

**Published:** 2026-10-23

**Authors:** Wei Shi, Hongjing Xu, Caihong Pu, Yuanzhong Liang, Ting Zhao, Xueli Wang, Zhenxing Liu, Chunhua Zhang, Ting Li, Fengquan He

**Affiliations:** ^1^ Clinical Laboratory Honghe Hani and Yi Autonomous Prefecture Maternal and Child Health Hospital Honghe Yunnan Province People's Republic of China; ^2^ Radiology Department Honghe Hani and Yi Autonomous Prefecture Maternal and Child Health Hospital Honghe Yunnan Province People's Republic of China; ^3^ Prenatal Diagnosis Department Honghe Hani and Yi Autonomous Prefecture Maternal and Child Health Hospital Honghe Yunnan Province People's Republic of China; ^4^ Ultrasound Department Honghe Hani and Yi Autonomous Prefecture Maternal and Child Health Hospital Honghe Yunnan Province People's Republic of China; ^5^ Department of Medical Genetics Yunnan Maternal and Child Health Hospital Kunming Yunnan Province People's Republic of China; ^6^ Department of Obstetrics Honghe Hani and Yi Autonomous Prefecture Maternal and Child Health Hospital Honghe Yunnan Province People's Republic of China

**Keywords:** 17p12 microdeletion, cerebral small vessel disease, COL4A1, fetal intracranial hemorrhage

## Abstract

A 33‐year‐old woman with two consecutive pregnancies affected by fetal left periventricular germinal matrix hemorrhage (Grade IV) and progressive ventriculomegaly underwent trio whole‐exome sequencing. A heterozygous COL4A1 c.4249G>C (p.Gly1417Arg) variant, classified as a variant of uncertain significance, was identified in the fetus of the current pregnancy and found to be maternally inherited; concurrent copy‐number analysis incidentally detected a maternal 17p12 microdeletion encompassing PMP22, which was not inherited by the fetus. Systematic reverse phenotyping of the mother revealed right corticospinal tract Wallerian degeneration, persistent microscopic hematuria, a renal cyst, and retinal arteriolar changes—a phenotype compatible with the COL4A1‐associated spectrum, although molecular causality remains unproven. Following multidisciplinary review, the pregnancy was terminated at 29 weeks, primarily on the basis of the grave fetal prognosis, with early‐onset preeclampsia contributing to the overall maternal risk assessment; the mother's post‐termination course was uneventful, with resolution of hypertension and proteinuria. This case illustrates that recurrent anatomically congruent fetal intracranial hemorrhages may prompt maternal reverse phenotyping that identifies maternally inherited COL4A1 variants of uncertain significance, and underscores the need to distinguish phenotypic concordance from proven molecular causality in prenatal counseling.

## Key Clinical Message

Recurrent fetal germinal matrix hemorrhage may prompt maternal reverse phenotyping and identification of a maternally inherited COL4A1 variant. When the variant is a VUS, prenatal decision‐making should rest on multidisciplinary assessment of fetal imaging and maternal phenotype, distinguishing phenotypic concordance from proven molecular causality.

## 1 Introduction

COL4A1, located at 13q34, encodes the alpha‐1 chain of Type IV collagen, which forms the core scaffold of vascular basement membranes in association with the alpha‐2 chain encoded by COL4A2 [1, 2]. Gould et al. first established that COL4A1 missense mutations cause autosomal dominant cerebral small vessel disease and hemorrhagic stroke [3]. The phenotypic spectrum of this condition—now designated Gould syndrome—encompasses fetal intracranial hemorrhage and porencephaly through to adult‐onset lacunar infarction and cognitive decline [4, 5].

Marked incomplete penetrance and variable expressivity characterize the disease; de novo mutations account for roughly 40% of cases [5]. Many carriers remain asymptomatic for years, with isolated microscopic hematuria or subtle ocular findings as the only manifestations [5, 6]. Consequently, affected women of reproductive age frequently escape diagnosis until severe fetal phenotypes prompt reverse phenotyping of the parent.

Fetal intracranial hemorrhage carries grave prognostic implications. Approximately 28% of severe fetal brain lesions harbor COL4A1/2 pathogenic variants [7], and over 60% of COL4A1/2 variant carriers develop ventriculomegaly or fetal growth restriction (FGR) in the second or third trimester [8]. Prenatal MRI enables early detection of porencephaly, schizencephaly, and other structural anomalies [9, 10].

We describe a case in which recurrent fetal intracranial hemorrhage prompted maternal reverse phenotyping that identified a maternally inherited COL4A1 variant of uncertain significance together with an incidental 17p12 microdeletion, and discuss the genetic diagnostic strategy and clinical management of a prenatal variant of uncertain significance (VUS).

## 2 Case Report

### 2.1 Clinical Data

A 33‐year‐old Han Chinese woman with three pregnancies presented with recurrent adverse pregnancy outcomes. The first pregnancy ended in biochemical loss, and the second and third were terminated at 27 and 29 weeks of gestation because of fetal cerebral anomalies. The parents were non‐consanguineous. In the second pregnancy, fetal brain anomalies detected at 22 weeks led to termination at 27 weeks for left ventriculomegaly and Grade IV germinal matrix hemorrhage; amniotic fluid karyotyping, CNV‐seq, and cytomegalovirus testing had all yielded normal results. The third pregnancy was conceived spontaneously. When fetal intracranial hemorrhage was detected at 23^+3^ weeks (Section 2.2), a targeted differential work‐up was initiated. Because fetal intracranial hemorrhage may result from fetal thrombocytopenia, coagulation disorders, or congenital infection, maternal platelet counts, coagulation profiles, and TORCH screening were performed and were unremarkable, and cytomegalovirus testing in the second pregnancy had likewise been negative. As fetal blood sampling and fetal‐specific hematological testing were not performed, fetal thrombocytopenia and fetal coagulation disorders could not be definitively excluded; however, none of these diagnoses was supported by the available investigations. The family pedigree is shown in Figure 1.

**FIGURE 1:**
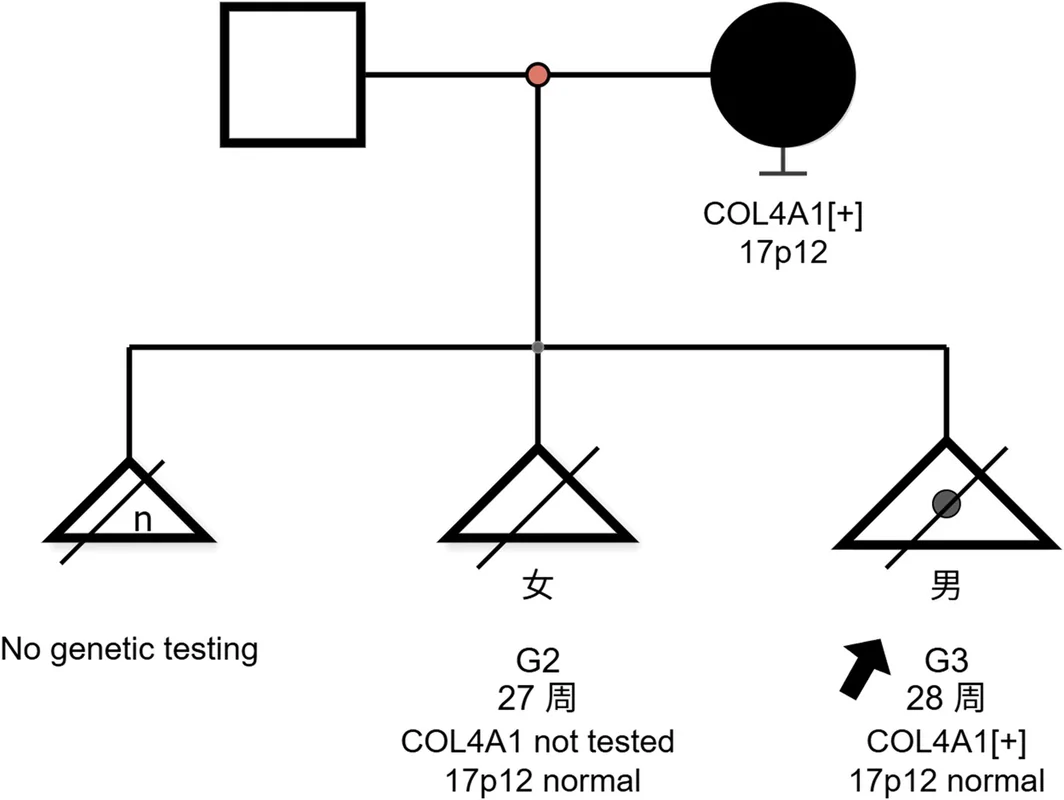
Pedigree of the family. Squares, males; circles, females; triangles, pregnancies; diagonal lines, pregnancy loss or termination. Filled circle: Affected mother (I‐2) carrying both the COL4A1 variant and the 17p12 microdeletion; open square: Unaffected father (I‐1); arrow: Proband (fetus of the third pregnancy, II‐3). The COL4A1 c.4249G>C (p.Gly1417Arg) variant was identified in the mother and proband fetus; the father was wild‐type. The 17p12 microdeletion was detected in the mother only and was not inherited by the proband fetus (II‐3) or by the fetus of the second pregnancy (II‐2). The fetus of the second pregnancy was investigated by karyotyping and CNV‐seq only and was not tested for the COL4A1 variant; the fetus of the first pregnancy (II‐1) underwent no genetic testing. “F”/”M” below triangles indicate female/male fetuses.

### 2.2 Fetal Imaging

The imaging findings were similar across the two pregnancies: Both demonstrated left periventricular germinal matrix hemorrhage (Grade IV) with progressive ipsilateral ventriculomegaly. Ventricular size was quantified as the left lateral ventricular width measured perpendicular to the glomus of the choroid plexus on coronal T2‐weighted images and graded according to the Society for Maternal‐Fetal Medicine classification (mild 10–12 mm, moderate 13–15 mm, severe > 15 mm) [11].

In the second pregnancy, fetal MRI at 22^+4^ weeks demonstrated Grade IV hemorrhage in the left periventricular germinal matrix with mild left ventriculomegaly (10.87 mm; Figure 2); follow‐up at 27^+4^ weeks showed partial hematoma resolution but progression to severe ventriculomegaly (21.36 mm; Figure 2).

**FIGURE 2:**
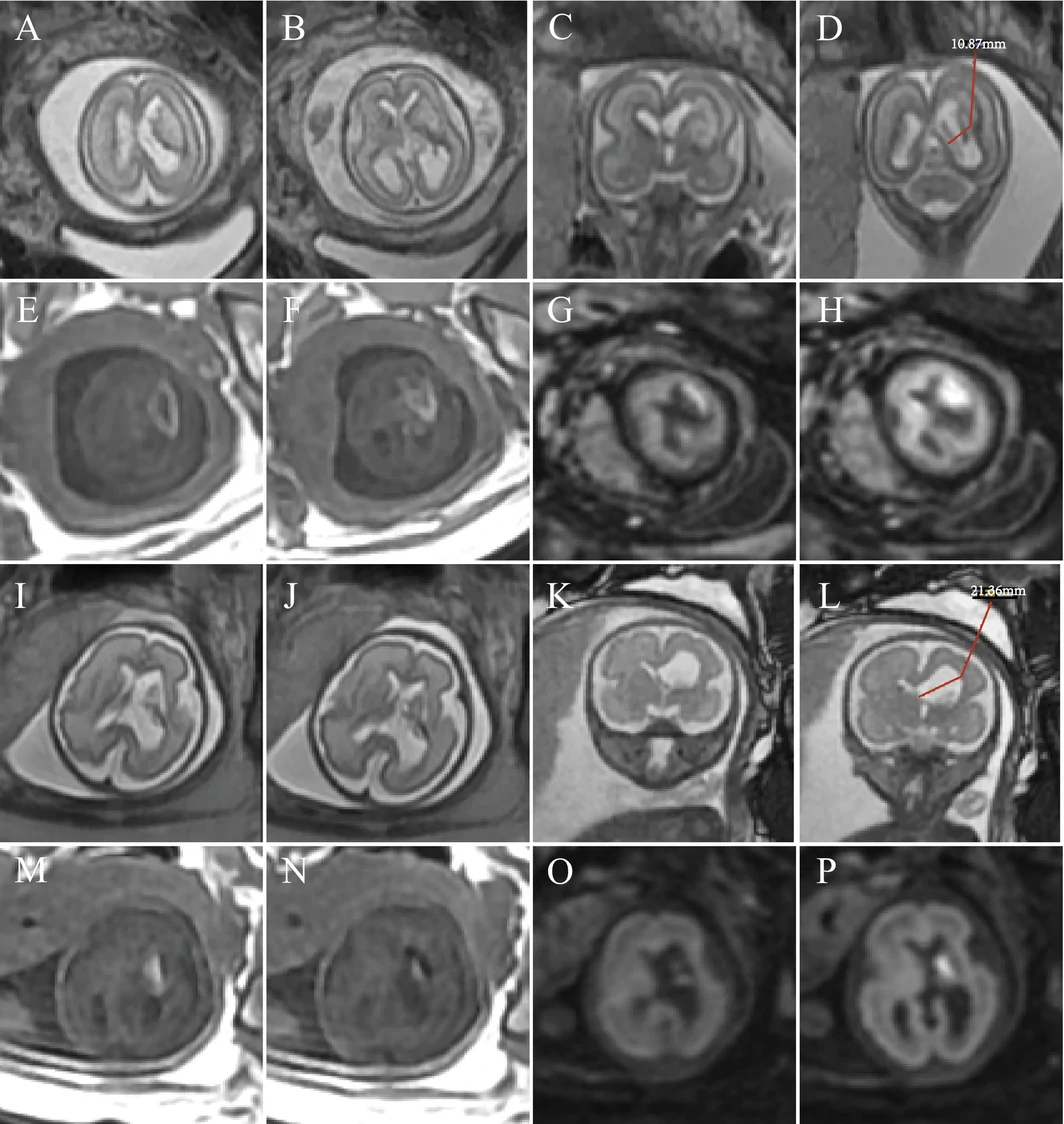
Fetal cranial MRI of the second pregnancy. (A–H) 22^+4^ weeks: Predominantly hyperintense T2 signal in the left periventricular germinal matrix and basal ganglia with mixed T1 signals and extensive DWI hyperintensity—Grade IV hemorrhage—with mild left ventriculomegaly (ventricular width 10.87 mm, coronal T2WI perpendicular to the choroid‐plexus glomus). (I–P) 27^+4^ weeks: Patchy residual subacute hemorrhage with progression to severe ventriculomegaly (21.36 mm).

In the current pregnancy, ultrasound at 23^+3^ weeks identified a mixed‐echogenicity mass in the region of the left choroid plexus (2.47 × 1.51 × 1.35 cm) with internal and peripheral vascularity on color Doppler, together with widening of the left lateral ventricle (Figure 3).

**FIGURE 3:**
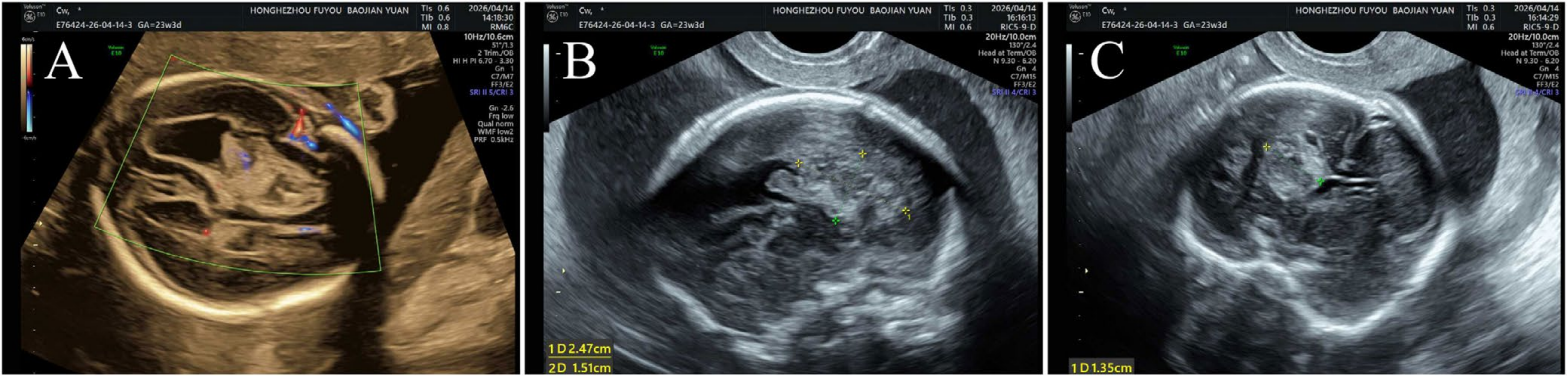
Prenatal ultrasound of the third pregnancy at 23^+3^ weeks. (A) Transventricular view: Mixed‐echogenicity mass in the region of the left choroid plexus (2.47 × 1.51 × 1.35 cm) with internal and peripheral vascularity on color Doppler, consistent with the intraventricular component of hemorrhage. (B, C) Coronal and additional views showing left lateral ventricular widening and the extent of intraventricular echogenic material. Note: An aberrant right subclavian artery and an echogenic intracardiac focus had been noted at 16 weeks.

Fetal MRI at 23^+4^ weeks confirmed Grade IV left periventricular germinal matrix hemorrhage with mild ventriculomegaly (10.47 mm; Figure 4). By 28^+0^ weeks, the hematoma had partially resolved, but the left ventricle had progressed to severe dilatation (15.96 mm; Figure 4). The sonographic choroid‐plexus mass corresponded to the intraventricular component of the hemorrhage, whereas MRI delineated its origin in the periventricular germinal matrix with extension into the basal ganglia; the two modalities therefore depicted different components of a single hemorrhagic process.

**FIGURE 4:**
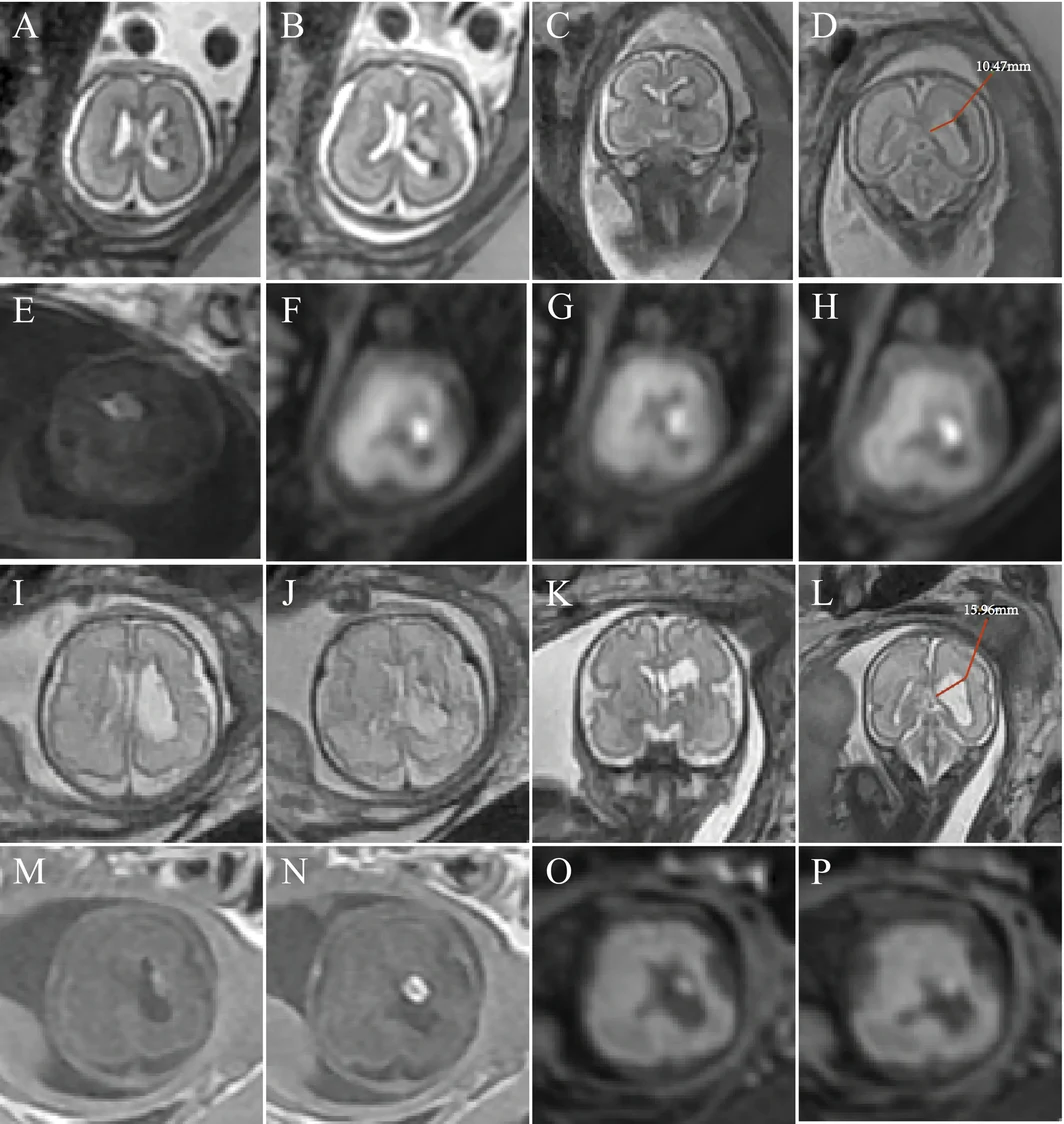
Fetal cranial MRI of the third pregnancy. (A–H) 23^+4^ weeks: Grade IV left periventricular germinal‐matrix hemorrhage with basal‐ganglia extension, patchy trigone T2 hypointensity, and mild ventriculomegaly (10.47 mm). (I–P) 28^+0^ weeks: Patchy residual hemorrhage with severe ventriculomegaly (15.96 mm); concurrent fetal growth restriction (biometry and percentiles in Section 2.2).

Concurrent fetal growth restriction was documented biometrically: The estimated fetal weight fell from 522 g (18th percentile) at 23^+3^ weeks to 857 g (0.2nd percentile) at 28^+3^ weeks, with the abdominal circumference (215 mm, 1.4th percentile), head circumference (244 mm, 1.1th percentile), and femur length (46 mm, 0.9th percentile) all below the 3rd percentile on the NICHD fetal growth reference. Umbilical artery Doppler velocimetry was not performed; the diagnosis of fetal growth restriction therefore rested on biometric criteria with a progressive percentile decline.

### 2.3 Maternal Phenotypic Evaluation

Systematic history‐taking and physical examination disclosed previously unrecognized multi‐system abnormalities compatible with the COL4A1‐associated spectrum (Table 1).

**TABLE 1:** Comparison of maternal multi‐system phenotypes in this case with reported COL4A1‐associated manifestations in the literature.

Organ system	Phenotype in the present case (maternal)	Reported COL4A1‐associated manifestations
Nervous system	Right corticospinal tract Wallerian degeneration; left lower limb claudication; left clubfoot; limb asymmetry	Motor impairment (23.6%), stroke (29.6%), epilepsy (29.0%), porencephaly/schizencephaly (24.5%) [7]
Renal	Persistent microscopic hematuria (occult blood 2+); right renal simple cyst (1.2 cm)	Hematuria (9.2%), renal cysts (feature of HANAC syndrome) [2, 12]
Ocular	Hyperopia/amblyopia/astigmatism; fundus A‐V nicking; retinal arterial tortuosity	Retinal arterial tortuosity (8.5%, up to 38.5% in oligosymptomatic carriers), anterior segment dysgenesis (30.8%), cataract (18.1%) [7]
Cranial MRI	Right corticospinal tract Wallerian degeneration; compensatory dilation of right lateral ventricle	Porencephaly, white matter lesions, lacunar infarcts, microbleeds [4, 5]
Other	Early‐onset preeclampsia	Gestational vascular complications (infrequently reported)

*Note:* Percentages are derived from Gasparini et al. [7] unless otherwise indicated.

Abbreviation: HANAC, Hereditary Angiopathy with Nephropathy, Aneurysms, and Muscle Cramps.

Neuromotor: Left lower‐limb claudication dating from childhood, long misattributed to poliomyelitis sequelae. Examination revealed left clubfoot deformity, bilateral leg‐length and girth asymmetry (left shorter and thinner), left upper‐limb weakness (Grade 4/5 versus 5/5 on the right), and left‐sided hypoesthesia. Cranial MRI (Figure 5) showed continuous unilateral volume loss from the right posterior limb of the internal capsule through the cerebral peduncle, pons, and medulla, consistent with chronic Wallerian degeneration of the right corticospinal tract. The right lateral ventricle was compensatorily dilated (maximum diameter 15.8 mm).

**FIGURE 5:**
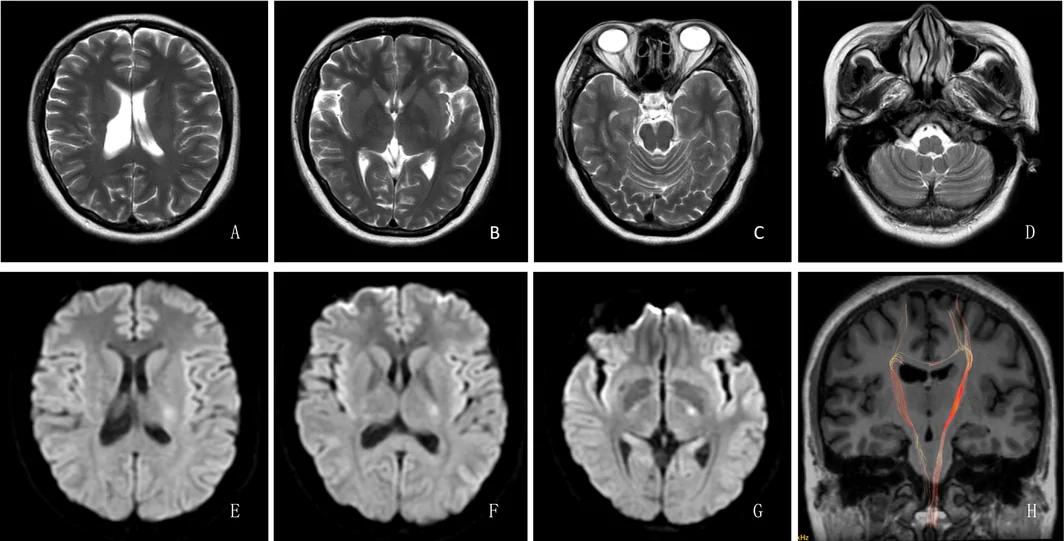
Maternal cranial MRI at approximately 24 weeks of gestation. (A–D) Axial T2WI: Right periventricular encephalomalacia with localized ventricular dilatation and atrophy of the right posterior limb of the internal capsule, right cerebral peduncle, and ventral medulla. (E–G) DWI: Reduced diffusion signal and volume loss of the right corticospinal tract. (H) Coronal T1WI with DTI tractography fusion: Decreased right corticospinal fiber density. Overall: Chronic right corticospinal tract Wallerian degeneration.

Renal: Persistent microscopic hematuria (urine occult blood 2+, RBC 15–20/HP) without proteinuria. Renal ultrasound demonstrated a 1.2‐cm simple cyst in the lower pole of the right kidney. Renal function and eGFR were normal.

Ocular: Bilateral hyperopia, amblyopia, and astigmatism (best‐corrected visual acuity: OD 0.6, OS 0.5). Funduscopy (Figure 6) revealed enhanced arteriolar reflexes, arteriovenous nicking, and localized retinal arteriolar tortuosity—all documented before the onset of preeclampsia.

**FIGURE 6:**
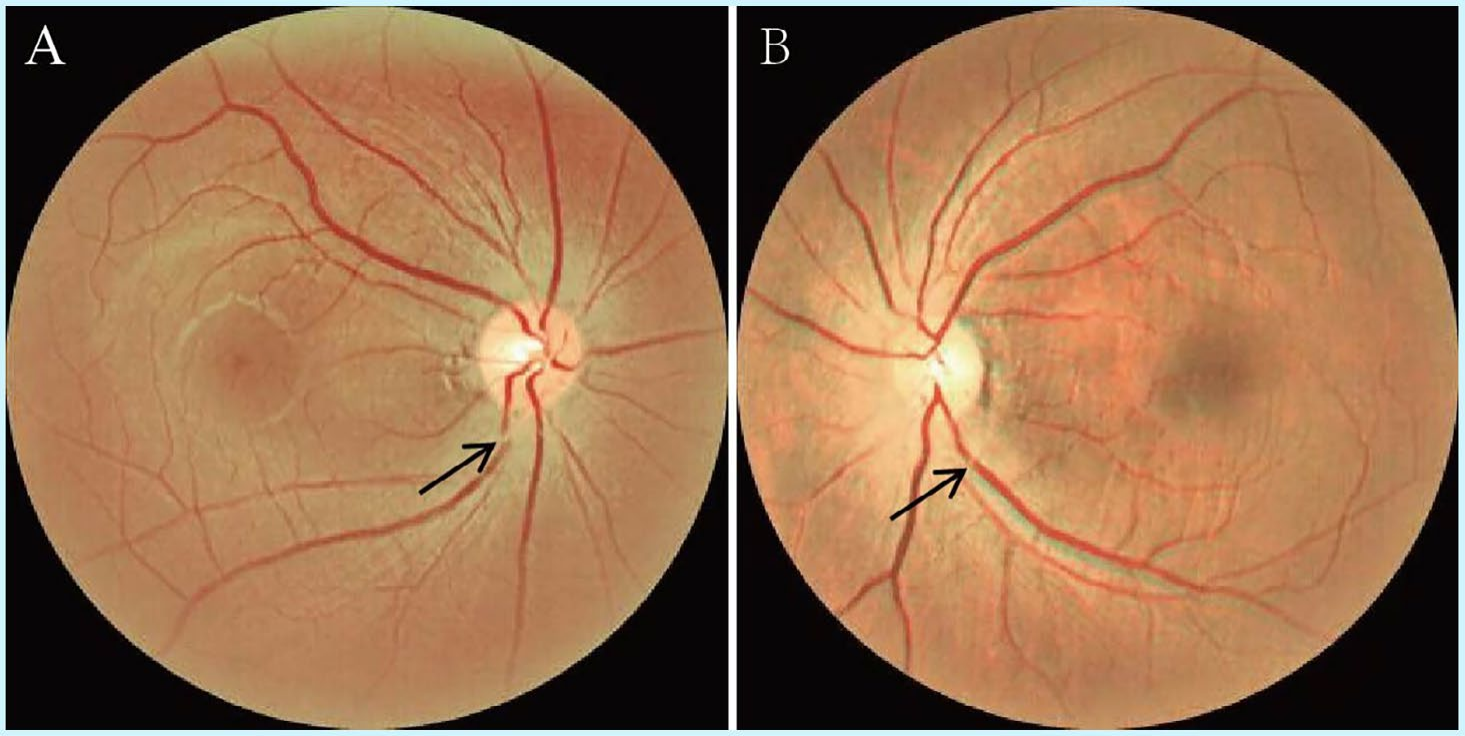
Maternal fundus photography. (A) Right eye: Enhanced arteriolar reflex and arteriovenous nicking (arrowheads). (B) Left eye: Localized retinal arteriolar tortuosity (arrows). Findings were documented before the onset of preeclampsia; a superimposed contribution from preeclampsia‐related hypertension cannot be entirely excluded.

Pregnancy complication: Early‐onset preeclampsia developed at 27^+^ weeks (blood pressure 150/85 mmHg, proteinuria ++), managed under high‐risk obstetric surveillance.

### 2.4 Genetic Testing

Previous amniotic fluid karyotyping (G‐banding) and CNV‐seq were unremarkable. Trio‐WES was performed on the mother, father, and fetal amniocytes (Illumina NovaSeq 6000 platform; target region coverage ≥ 99%, mean sequencing depth ≥ 100 ×).

#### 2.4.1 COL4A1 Variant Detection and Validation

WES identified a heterozygous missense variant, COL4A1 (NM_001845.6) c.4249G>C (p.Gly1417Arg), in the fetus and mother; the father was wild‐type. Sanger sequencing confirmed co‐segregation (Figure 7). Located in exon 48, the variant disrupts a highly conserved glycine within the Gly‐X‐Y motif of the collagen triple helix. In silico prediction tools (REVEL 0.977, SIFT 0.00, PolyPhen‐2 1.000) support a deleterious effect; the allele frequency is zero in gnomAD v4.0 East Asian. ACMG/AMP classification—PM1 (critical functional domain), PM2_Supporting (absent from population databases), and PP3 (computational evidence)—yielded a VUS (Table 2). De novo status could not be assessed, and the variant is absent from ClinVar and HGMD. Of note, the fetus of the second pregnancy had been investigated by karyotyping and CNV‐seq only and was not tested for the COL4A1 variant, which was identified after the third pregnancy; this limitation is addressed in the Discussion.

**FIGURE 7:**
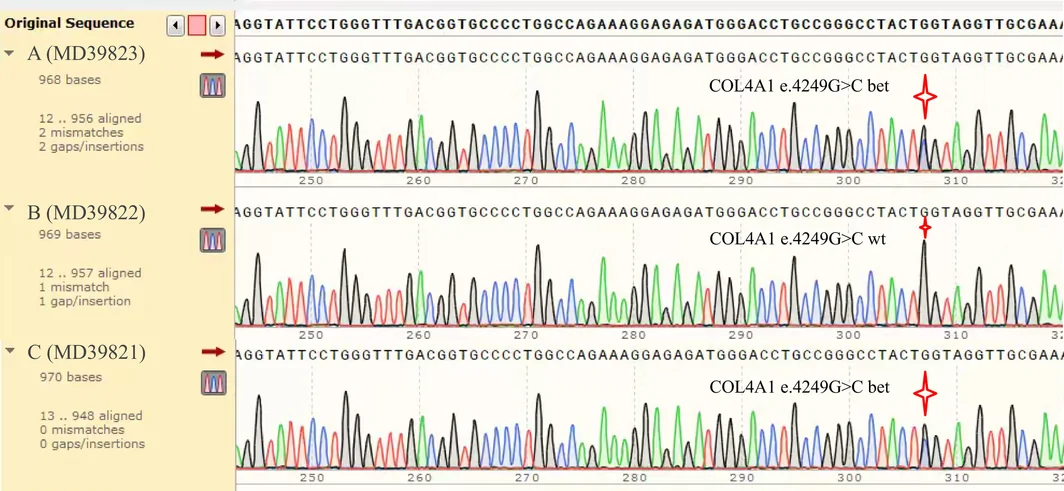
Sanger sequencing of COL4A1 c.4249G>C (p.Gly1417Arg). (A) Fetal amniocyte DNA: Heterozygous variant. (B) Paternal DNA: Wild‐type. (C) Maternal DNA: Heterozygous variant, consistent with maternal inheritance. The variant was absent from ClinVar and HGMD at submission.

**TABLE 2:** Summary of ACMG/AMP evidence for the COL4A1 c.4249G>C (p.gly1417Arg) variant.

Evidence code	Evidence type	Specific basis
PM1	Located in a critical functional domain/critical functional domain	Collagen triple helix domain (exon 48); disrupts a glycine residue within the highly conserved Gly‐X‐Y motif [2]
PM2_Supporting	Absent from population frequency databases	gnomAD v4.0 East Asian population allele frequency = 0; not recorded in ClinVar or HGMD
PP3	Computational evidence supports a deleterious effect	REVEL = 0.977; SIFT = 0.00 (deleterious); PolyPhen‐2 = 1.000 (probably damaging)
**Overall classification**		**Variant of Uncertain Significance (VUS)**

*Note:* De novo evidence (PS2/PM6) could not be evaluated due to the unavailability of grandparental samples. PM5 could not be applied as the variant is absent from curated mutation databases.

Abbreviation: ACMG/AMP, American College of Medical Genetics and Genomics/Association for Molecular Pathology.

#### 2.4.2 17p12 Microdeletion Detection and Validation

WES‐CNV analysis (ExomeDepth v1.1.10) revealed an ~1.33 Mb heterozygous deletion at 17p12 (chr17:g.14084952_15414065del, GRCh37) in the mother, encompassing the entire PMP22 gene. This deletion was independently validated by low‐pass CNV‐seq (Figure 8). The region carries a ClinGen haploinsufficiency score of 3 and is associated with hereditary neuropathy with liability to pressure palsies (HNPP). Fetal CNV‐seq confirmed that the deletion was not inherited.

**FIGURE 8:**
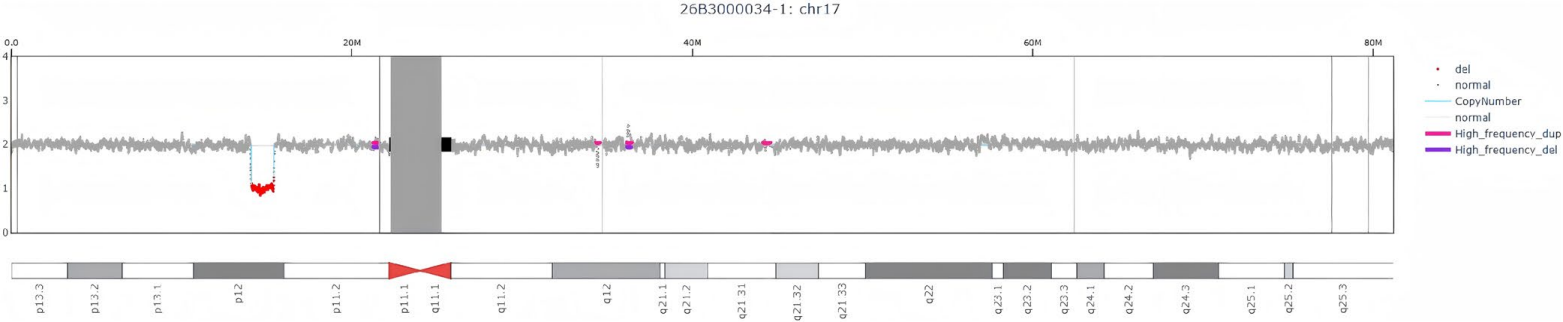
CNV‐seq confirmation of the maternal 17p12 microdeletion. Upper panel: Log_2_ ratio reduction spanning ~1.33 Mb at 17p12 (chr17:G.14084952_15414065del, GRCh37), encompassing PMP22 (red bar; ClinGen haploinsufficiency score 3, HNPP). Lower panel: Gene map of the deleted region. Fetal CNV‐seq confirmed that the deletion was not inherited (data not shown).

### 2.5 Clinical Integration and Pregnancy Outcome

Pre‐test genetic counseling had explicitly addressed the possibility of secondary findings. After the 17p12 deletion was identified, dedicated counseling was provided according to ACMG guidelines, and the uncertainty surrounding the COL4A1 VUS was communicated transparently.

The multidisciplinary review (prenatal diagnosis, neonatology, ultrasound, radiology) considered the fetal and maternal indications separately. Regarding the fetus, Grade IV periventricular hemorrhage with progressive severe ventriculomegaly and early‐onset fetal growth restriction carried a high risk of severe neurodevelopmental impairment irrespective of the genetic findings; the COL4A1 variant, as a VUS, was not itself used as an indication. Regarding the mother, early‐onset preeclampsia together with the cerebral and retinal small‐vessel changes identified on reverse phenotyping raised concern that continued gestation could add to her vascular burden; however, given the VUS classification, any contribution of the COL4A1 variant to maternal cerebrovascular risk remains uncertain and was not presented as an established risk. No curative intrauterine intervention exists. After informed consent, the couple elected termination at 29 weeks; fetal autopsy was declined. The mother's post‐termination course was uneventful, with resolution of hypertension and proteinuria; she continues routine outpatient follow‐up.

For future pregnancies, the probability of inheriting each variant is 50% per pregnancy independently (25% probability of co‐inheritance); because the COL4A1 variant remains a VUS and COL4A1‐related disorders show incomplete penetrance, these inheritance probabilities should not be equated with disease‐risk estimates. Options include chorionic villus sampling or amniocentesis with COL4A1 testing and CNV‐seq. PGT‐M could establish whether an embryo inherits the variant but would not establish its pathogenicity; its availability and utility would require case‐specific assessment by a reproductive genetics service. A chronological summary of key clinical events is presented in Table 3.

**TABLE 3:** Chronological summary of clinical events.

Pregnancy/gestational age	Event	Key findings/outcome
Second, 22^+4^ wks	Fetal MRI	Grade IV left periventricular GMH; mild ventriculomegaly (10.87 mm)
Second, 27^+4^ wks	Follow‐up MRI; termination	Partial hematoma resolution; severe ventriculomegaly (21.36 mm); karyotype/CNV‐seq/CMV normal
Third, 16 w	Ultrasound	Aberrant right subclavian artery; echogenic intracardiac focus
Third, 23^+3^ wks	Ultrasound	Mixed‐echogenicity left choroid‐plexus mass (2.47 × 1.51 × 1.35 cm)
Third, 23^+4^ wks	Fetal MRI	Grade IV left periventricular GMH; mild ventriculomegaly (10.47 mm)
Third, 27^+4^ wks	Maternal complication	Early‐onset preeclampsia (BP 150/85 mmHg, proteinuria ++)
Third, 28^+0^/28^+3^ wks	Follow‐up MRI; biometry	Partial hematoma resolution; severe ventriculomegaly (15.96 mm); EFW 857 g (0.2nd percentile)
Third, 29 w	MDT review; termination	Decision based primarily on fetal prognosis; uneventful maternal recovery
Future	Reproductive counseling	CVS/amniocentesis with COL4A1 testing + CNV‐seq; PGT‐M requires case‐specific assessment

Abbreviations: GMH, germinal matrix hemorrhage; MDT, multidisciplinary team; PGT‐M, preimplantation genetic testing for monogenic disorders.

## 3 Discussion

Three principal observations emerge from this case.

Recurrent fetal hemorrhage as a clue prompting maternal evaluation. Recurrent fetal intracranial hemorrhage—particularly when anatomically concordant across pregnancies—should prompt consideration of an inherited maternal vascular predisposition and systematic reverse phenotyping of the mother. The periventricular germinal matrix is inherently vulnerable to basement‐membrane disruption, and recurrence favors a genetic contribution over sporadic bleeding [7, 8]. In this family, the mother's cerebral, renal, and retinal findings are compatible with the COL4A1‐associated spectrum and strengthen the phenotypic concordance, although they do not establish molecular causality. The left‐sided laterality observed in both pregnancies is more plausibly attributable to stochastic hemodynamic variation than to a true lateralizing genetic effect, and maternal preeclampsia may have acted as an environmental modifier amplifying fetal hemodynamic stress. This observation supports incorporating maternal pregnancy complications into the overall assessment of COL4A1‐associated fetal brain injury.

Value of integrated WES–CNV analysis. Performing CNV analysis in parallel with WES identified the maternal 17p12 deletion, which would have been missed by single‐nucleotide variant analysis alone. Because the deletion was not inherited by the fetus and does not explain the fetal hemorrhage, its contribution in this case lies in refining the maternal phenotype and informing reproductive counseling rather than in resolving the prenatal presentation. The maternal 17p12 deletion (HNPP) does not account for her central motor deficit, which is anatomically explained by corticospinal tract degeneration. Each variant segregates independently, yielding a 50% per‐pregnancy inheritance probability for each and a 25% probability of co‐inheritance—probabilities that must be distinguished from disease‐risk estimates, given the VUS status and the incomplete penetrance of COL4A1‐associated disorders.

Managing a prenatal VUS. The p.Gly1417Arg substitution resides at a C‐terminal glycine within the collagen triple helix, a region previously associated with severe cerebrovascular phenotypes [2, 13]. Despite phenotypic concordance across two generations, strict application of ACMG/AMP criteria yields a VUS because de novo evidence and database entries are lacking. Crucially, the termination decision was not predicated on the VUS: The fetal indication—Grade IV hemorrhage with progressive severe ventriculomegaly and early‐onset fetal growth restriction—carried a grave neurodevelopmental prognosis independent of the genetic result, whereas the maternal contribution was considered cautiously and communicated as uncertain. This separation of evidence classification from clinical reasoning aligns with contemporary MDT‐anchored frameworks for prenatal VUS management [14, 15, 16], and we emphasize the importance of transparently communicating scientific uncertainty to families.

Limitations. The family declined fetal autopsy, precluding ultrastructural verification of basement‐membrane abnormalities. The fetus of the second pregnancy was not tested for the COL4A1 variant; although the imaging pattern was similar across the two pregnancies, attribution of both hemorrhages to the same variant therefore remains presumptive. In vitro functional validation of the COL4A1 variant is ongoing through a collaborative cell‐model project. EMG/NCV studies were not performed, representing the main gap in quantifying peripheral nerve involvement attributable to the 17p12 deletion. Because the fetus did not inherit the 17p12 deletion, this case cannot address potential synergistic effects of dual genetic burden. Umbilical artery Doppler data were not available; fetal growth restriction was defined biometrically. Finally, although the termination decision was grounded in comprehensive phenotyping and MDT review, the VUS classification of the COL4A1 variant means that this approach lacks direct endorsement under current ACMG guidelines; broader generalization will require validation in additional families.

## 4 Conclusion

Recurrent fetal germinal‐matrix hemorrhage may prompt maternal reverse phenotyping that identifies maternally inherited COL4A1 variants of uncertain significance. In such cases, prenatal decision‐making should be guided by multidisciplinary assessment of fetal imaging and the observed maternal multi‐system phenotype, with phenotypic concordance clearly distinguished from proven molecular causality. Integrated WES with concurrent CNV analysis may aid characterization of complex prenatal presentations and reproductive counseling. However, definitive attribution of the variant to the fetal phenotype awaits functional validation and segregation in additional families.

## Author Contributions

**Wei Shi:** conceptualization, methodology, writing – review and editing, writing – original draft, visualization, project administration, software, formal analysis. **Hongjing Xu:** software, investigation, conceptualization, formal analysis, supervision, writing – original draft. **Caihong Pu:** conceptualization, investigation, writing – original draft, software, methodology, formal analysis. **Yuanzhong Liang:** visualization, software, formal analysis, validation. **Ting Zhao:** software, formal analysis, visualization, validation. **Xueli Wang:** conceptualization, validation, visualization, supervision, data curation. **Zhenxing Liu:** software, formal analysis, validation, visualization. **Chunhua Zhang:** software, data curation, visualization, investigation. **Ting Li:** writing – original draft, writing – review and editing, project administration, funding acquisition, conceptualization, methodology. **Fengquan He:** conceptualization, methodology, writing – original draft, writing – review and editing, resources, project administration, funding acquisition.

## Funding

The authors have nothing to report.

## Ethics Statement

This study was approved by the Ethics Committee of Honghe Hani and Yi Autonomous Prefecture Maternal and Child Health Hospital (Approval No. (2026) Hongfu You Lun Shen No. 16).

## Consent

Written informed consent was obtained from the pregnant woman and her spouse.

## Conflicts of Interest

The authors declare no conflicts of interest.

## Data Availability

The data that support the findings of this study are available on request from the corresponding author. The data are not publicly available due to privacy or ethical restrictions.
